# The identification of the anthracycline aclarubicin as an effective cytotoxic agent for pancreatic cancer

**DOI:** 10.1097/CAD.0000000000001283

**Published:** 2022-03-23

**Authors:** Thomas P. Brouwer, Sabina Y. van der Zanden, Manon van der Ploeg, Jaap D.H. van Eendenburg, Bert A. Bonsing, Noel F.C.C. de Miranda, Jacques J. Neefjes, Alexander L. Vahrmeijer

**Affiliations:** Departments of a Surgery; b Pathology; c Cell and Chemical Biology, Leiden University Medical Center, Leiden; d Oncode Institute, The Netherlands

**Keywords:** aclarubicin, chemo resistance, chemotherapy, epigenetic modification, pancreatic ductal adenocarcinoma, pancreatic ductal adenocarcinoma

## Abstract

Pancreatic ductal adenocarcinoma (PDAC) is one of the most lethal types of cancer, mainly due to its delayed diagnosis and lack of effective therapeutic options. Therefore, it is imperative to find novel treatment options for PDAC. Here, we tested a series of conventional chemotherapeutics together with anthracycline compounds as single agents or in combination, determining their effectivity against established commercial and patient-derived, low-passage PDAC cell lines. Proliferation and colony formation assays were performed to determine the anticancer activity of anthracyclines; aclarubicin and doxorubicin, on commercial and patient-derived, low-passage PDAC cell lines. In addition, the effect of standard-of-care drugs gemcitabine and individual components of FOLFIRINOX were also investigated. To evaluate which mechanisms of cell death were involved in drug response, cleavage of poly(ADP-ribose)polymerase was evaluated by western blot. Aclarubicin showed superior antitumor activity compared to other anthracyclines and standard of care drugs (gemcitabine and individual components of FOLFIRINOX) in a patient-derived, low-passage PDAC cell line and in commercial cell lines. Importantly, the combination of gemcitabine and aclarubicin showed a synergistic effect at a dose range where the single agents by themselves were ineffective. In parallel, evaluation of the antitumor activity of aclarubicin demonstrated an apoptotic effect in all PDAC cell lines. Aclarubicin is cytotoxic for commercial and patient-derived low-passage PDAC cell lines, at doses lower than peak serum concentrations for patient treatment. Our findings support a (re)consideration of aclarubicin as a backbone of new combination regimens for pancreatic cancer patients.

## Introduction

Pancreatic ductal adenocarcinoma (PDAC) is currently the seventh most lethal cancer type worldwide and, by 2025, is expected to become the third most lethal cancer type [1]. Throughout the years, most cancer patients have benefited from innovative therapeutic options that translated into improved survival; however, treatment options are still ineffective for most PDAC patients with the 5-year overall survival of PDAC patients lingering at around 8% [2]. First-line chemotherapy with gemcitabine (in combination with nab-paclitaxel or capecitabine) or FOLFIRINOX (5-FU, leucovorin, irinotecan, and oxaliplatin), have had little impact on overall survival in both neoadjuvant and adjuvant settings [3–5]. FOLFIRINOX treatment was reported to improve the overall survival compared to gemcitabine in patients with (metastasized) pancreatic cancer [6,7]. However, the slight improvement in survival with this treatment strategy coincided with a significant increase in toxicity [6]. These disappointing statistics underline the need for novel therapeutic strategies and associated predictive biomarkers for the treatment of pancreatic cancer.

A growing emphasis of drug discovery efforts for the treatment of malignancies has been on targeting the epigenome, including DNA methylation and histone modifications [8,9]. Epigenetic dysregulation, such as enrichment for certain histone-associated modifications, has emerged as a critical factor for tumorigenesis and metastasis [10–12]. Most histone modifying enzymes exhibit specificity toward particular histones or histone modifications, thus constituting ideal targets to develop cancer therapies [13]. Several epigenetic inhibitors are already being tested in combination with conventional chemotherapy as well as immunotherapies [14,15]. Aclarubicin and its well-known analog doxorubicin are anthracyclines, with doxorubicin being extensively used in the clinic to treat a variety of cancer types [16]. The main mechanism of anticancer activity by anthracyclines is considered to be the interference with Topoisomerase (Topo) IIα activity resulting in double-stranded DNA breaks [17]. In proliferating cells, this activity can result in mitotic catastrophe and cellular death [18]. However, whether this is the main cytotoxic activity of anthracyclines was challenged by the discovery of aclarubicin’s mode of action that does not involve double-stranded DNA breaks. It was recently shown that doxorubicin and aclarubicin also induce chromatin damage by evicting histones at discrete genomic regions [19,20]. While doxorubicin preferentially evicts histones from open chromatin regions, aclarubicin evicts histones from H3K27me3-marked heterochromatin [19–21]. Besides, in comparison to doxorubicin, aclarubicin does not induce DNA breaks but only induces histone eviction [19]. So far, aclarubicin has not been extensively tested, (pre) clinically, in solid cancers, let alone PDAC tumors. In fact, aclarubicin is not available for clinical testing beyond Japan and China, where it is mainly used for the treatment of hematological tumors [22]. A potential advantage of aclarubicin is the reduced cardiotoxicity and limited toxicity on reproductive organs, common side effects of other chemotherapies including doxo- and daunorubicin, mitoxantrone, and 5-FU [23,24]. Furthermore, as cardiotoxicity is treatment limiting, aclarubicin may be used in combination with other drugs or with more extensive treatment regimens compared to other chemotherapeutics [25].

In the present study, we performed a drug screening for a series of cytotoxic anticancer drugs using patient-derived, low-passage PDAC cell cultures and commercial PDAC cell lines. We demonstrate a superior anticancer activity of the compound aclarubicin, thereby illustrating the potential of this anthracycline for the treatment of PDAC. In addition, aclarubicin showed strong synergistic effects when combined with gemcitabine, a compound currently being used in pancreatic cancer treatment.

## Methods

### Patient and tissue specimens

Tumor tissue was collected from two PDAC patients at the Leiden University Medical Centre (LUMC) in accordance with Institutional Review Board (IRB) protocols (protocol P17.047). All specimens were anonymized and handled according to the ethical guidelines described in the Code for Proper Secondary Use of Human Tissue of the Netherlands of the Dutch Federation of Medical Scientific Societies and in accordance with the declaration of Helsinki. For the generation of low-passage pancreatic cancer cell cultures, fresh tumor tissue was processed as follows: resection material was collected in a sterile conical tube containing Iscove Modified Dulbecco Media + (IMDM+) GlutaMAX media (Thermo Fischer Scientific, Waltham, Massachusetts) with 10% Fetal Calf Serum (FCS) (Sigma-Aldrich, Saint Louis, Missouri) and 1% penicillin/streptomycin (Thermo Fisher Scientific), 1% Fungizone (Thermo Fisher Scientific), 0.1% Ciprofloxacin (provided by the LUMC pharmacy), and 0.1% Gentamicin (Sigma-Aldrich) on wet ice during transport from the operating room to the research laboratory. Upon arrival, a specimen from the resection material was manually minced using a sterile scalpel. The specimens underwent an overnight, enzymatic digestion step with 1 mg/mL collagenase (Sigma-Aldrich) and 1 mg/mL dispase (Invitrogen, Carlsbad, California) in 3 mL Dulbecco’s Modified Eagle Medium (DMEM) with 2% GlutaMAX (Thermo Fischer Scientific), at room temperature. After manual shaking to disintegrate bigger fragments, RPMI 1640 (Thermo Fischer Scientific) was added up to 50 mL and the mix was subsequently spun down for 5 min at 1500 rpm. After several washing steps with RPMI 1640, cells were resuspended in DMEM/F12 (Thermo Fisher Scientific) and DMEM, supplemented with 10% FCS (Thermo Fisher scientific) (mix 1:1) and split into separate wells on a 24-wells plate and placed at 37 °C. Patient-derived tumor cell cultures were refreshed once a week with the cell culture mix containing DMEM/F12 (Thermo Fisher Scientific) and DMEM, supplemented with 10% FCS until tumor outgrowth was observed.

### Cell culture

PDAC cell lines were maintained in DMEM/F12 (Gibco) and DMEM 1x + Glutamax -1 (Gibco), supplemented with 10% FCS. BXPC-3, CAPAN-2, and CFPAC-1 cells were obtained from ATCC (www.ATCC.org). Their identity was confirmed by using STR profiling (*GenePrint* 10 system, Promega), and kept under low passage. The in-house primary human PDAC lines, PC25 and PC54, were also typed by STR sequencing during the screens, and kept under low passage afterward. All cell lines were maintained in a humidified atmosphere at 5% CO_2_ at 37 °C and regularly tested for the absence of mycoplasma.

### Proliferation assays

Cells were seeded into 96-well plates (2000–5000 cells per well). Twenty-four hours after seeding, cells were exposed for 4 h to the indicated drugs. Subsequently, drugs were removed and cells were washed with PBS, to model normal pharmacokinetics in the human body. Cell viability was measured 72 h posttreatment using the Cell Titer Blue viability assay (Promega). Fluorescence signal was measured using a Clariostar (BMG labtech) microplate reader. Relative survival was normalized to the untreated control and corrected for background signal.

### Colony formation assays

Cells were seeded into 12-well plates (1000–5000 cells per well). The next day, cells were treated for 2 h with the different drugs at indicated concentrations. Subsequently, drugs were removed and cells were left to grow for 7–12 days. Cells were fixed in 3.7% formaldehyde/PBS, and stained using 0.1% Crystal violet solution (Sigma). Images of the cell colonies were taken using Gelcount (Oxford Optronix). Analysis of colonies was done by ImageJ.

### Western blotting

Half million cells were seeded 24 h prior to treatment in a 12-well dish. Drug treatments were carried out for 2 h with the same dosages (10 µM) employed in the proliferation assays. Drugs were removed and the cells were cultured until the indicated time points (0, 12, and 24 h). Subsequently, cells were lysed directly in SDS-sample buffer (2% SDS, 10% glycerol, 5% β-mercaptoethanol, 60 mM Tris HCl pH 6.8, and 0.01% bromophenol blue). Lysates were resolved by SDS/polyacrylamide gel electrophoresis followed by Western blotting. Primary antibodies used for protein detection were anti-poly (ADP-ribose) polymerase (PARP) (1:1000, #9542, CST) and alpha-tubulin (1:3000, #3873, CST). Images were analyzed with ImageJ.

### Statistical analysis

All experiments were performed at least three times in an independent manner, unless otherwise specified. All data are presented as means ± SD. The results were analyzed using an unpaired, two-tailed, Student’s t-test. Statistical testing and graphical visualization were done with PRISM Graphpad software (version 8).

## Results

### Anthracyclines in pancreatic ductal adenocarcinoma

**Fig. 1:**
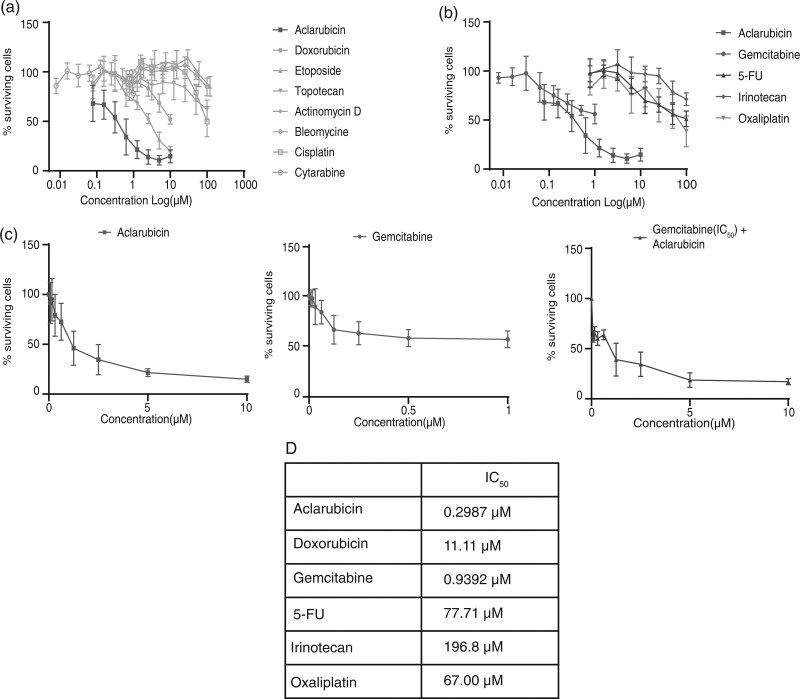
Cytotoxic effect of standard of care and anthracycline drugs in primary patient-derived cell lines. (a–c) Cells were treated for 4 h with the indicated drugs and cell viability was analyzed 72 h posttreatment using a CellTiter-Blue assay. Data are normalized to untreated cells and shown as mean ± SD. (d)Overview of the IC50 values.

Initially, the anticancer activity of several chemotherapeutics (Table 1), including a range of Topo IIα interfering agents, were tested in a low-passage cell line generated from a PDAC patient, PC25 (Table 2). Proliferation assays revealed that cellular fitness was differentially affected by the distinct chemotherapeutic compounds. Strikingly, aclarubicin, showed the most potent cytotoxic effect (Fig. 1a). Standard-of-care drugs (gemcitabine and individual components of FOLFIRINOX) failed to reach the same effect at comparable concentrations (Fig. 1b).

**Table 1:** Concentration range of the different chemotherapeutical compounds used for the proliferation and colony formation assays

Drugs	Range, µM	Company
Aclarubicin	0.078–10	Santa Cruz
Doxorubicin	0.078–10	Accord Healthcare limited
Etoposide	0.938–120	Pharmachemie
Topotecan	0.78–100	Accord Healthcare limited
Actinomycin D	0.078–10	Santa Cruz
Bleomycin	0.78–100	Eureco-Pharma
Cisplatin	0.78–100	Accord Healthcare limited
Cytarabine	0.0078–1	Accord Healthcare limited
Gemcitabine	0.0078–1	Actavis
5-FU	0.78–100	Accord Healthcare limited
Irinotecan	0.78–100	Fresenius Kabi
Oxaliplatin	0.78–100	Fresenius Kabi

**Table 2:** Patient characteristics of the patient-derived low-passage PDAC cell lines

	PC25	PC54
Age, years	72	64
Type of surgery	Total pancreatectomy	Distal pancreaticosplenectomy
Tumor classification	PDAC	PDAC
Survival, months	11 (adjuvant therapy)	13 (adjuvant therapy)
Derivation	Primary tumor	Primary tumor

PDAC, pancreatic ductal adenocarcinoma.

Anthracyclines as monotherapies have, so far, failed to make a clinical impact in PDAC patients. Therefore, we decided to test the cytotoxic effect of combining gemcitabine as the most frequently administered drug for PDAC patients with aclarubicin, the most potent anthracycline determined in our experiments. As expected, the effect of gemcitabine was augmented by aclarubicin in the combinatorial setting (Fig. 1c).

**Fig. 2:**
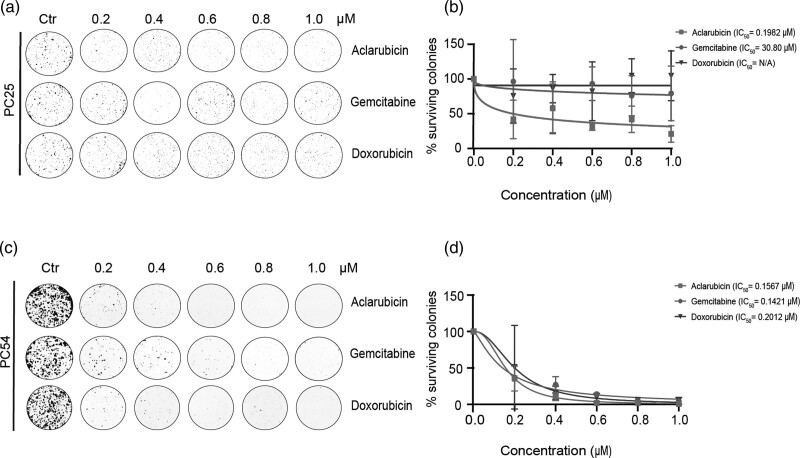
Aclarubicin retains superior anticancer effect using colony formation assays. (a–d)Colony formation assay for PC25 (a) and PC54 (c) cells treated for 2 h with indicated drugs. Percentage of surviving colonies is plotted per drugs for PC25 (b) and PC54 (d) as mean ± SD. Cell viability was normalized to untreated cells.

To validate these observations, in a subsequent experimental setting, colony forming assays were performed in the presence of gemcitabine, aclarubicin, and doxorubicin using PC25 and a second patient-derived, low-passage PDAC cell line, PC54 (Table 2). Concentrations of the different chemotherapeutical compounds were adjusted to the IC_50_ of gemcitabine (1 µM, Fig 1d). As in the proliferation assays, a marked decrease in colony formation in PC25 was observed when cells had been exposed to aclarubicin (Fig. 2a and b). Furthermore, in line with the proliferation assays, PC25 cells were insensitive to gemcitabine and doxorubicin. In the colony formation assays using PC54 cells, all three compounds decreased the formation of colonies with similar efficacy, although this cell line was characterized by a slow proliferation rate in culture (Fig. 2c and d). Altogether, aclarubicin demonstrated superior anticancer activity in low-passage patient-derived pancreatic cell lines, especially in the PC25 cell line that displayed resistance to gemcitabine and doxorubicin treatment.

### Superior antitumor effect of aclarubicin in pancreatic ductal adenocarcinoma cell lines

**Fig. 3:**
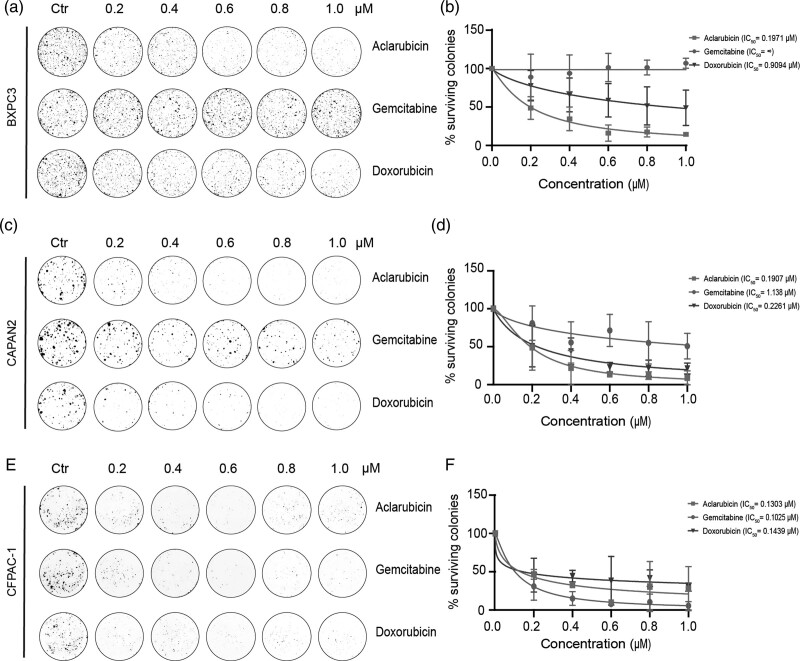
Aclarubicin effectivity in PDAC tumor cell lines. (a–f) Colony formation assay for BXPC-3 (a), CAPAN-2 (b) and CFPAC-1 (e). Cells were treated for 2 h with indicated drugs. Percentage of surviving colonies is plotted per drugs for BXPC-3 (b), PC54 (d) and CFPAC-1 (f). Normalized data is shown as mean ± SD.

To further expand our observations, we tested the effect of aclarubicin on the commercial cell lines BXPC-3, CAPAN-2, and CFPAC-1, in comparison to gemcitabine and doxorubicin. Intriguingly, the colony formation assays showed variable anticancer effects within the respective cell lines. Aclarubicin showed superior antitumor activity in two out of the three cell lines: BXPC-3 and CAPAN-2 (Fig. 3a–d), and was equally potent in the third cell line, CFPAC-1 (Fig. 3e–f). Contrary to previous research, BXPC-3 did not seem to be susceptible to gemcitabine treatment [26] but revealed high sensitivity to aclarubicin, as previously observed for the patient-derived cell line PC25. Similarly, CAPAN-2 was considerably more sensitive to treatment with both anthracyclines (aclarubicin and doxorubicin) than to the standard-of-care drug gemcitabine. All three drugs had a similar impact on CFPAC-1 when using colony formation assays. Of note, and similarly to PC54, the slow doubling time of the CFPAC-1 cell line considerably affected the read-out of colony formation. Nevertheless, these results demonstrate that different PDAC cell lines display distinct sensitivity to the drugs tested with aclarubicin being superior or at least equipotent to the other drugs tested.

### Apoptosis induction of pancreatic ductal adenocarcinoma cells by aclarubicin

**Fig. 4:**
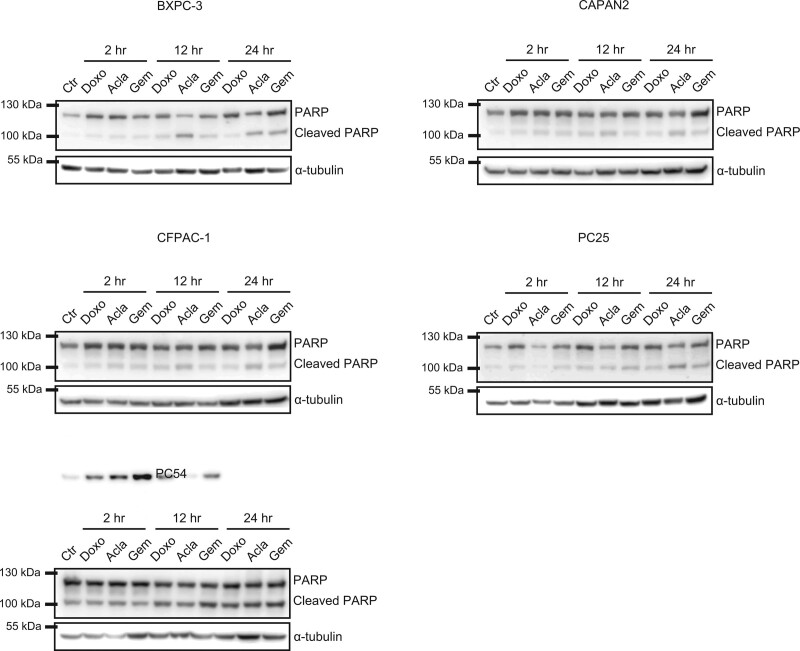
Induction of apoptosis by aclarubicin: Western blot assays looking into poly(ADP-ribose)polymerase (PARP) in the different cell lines at defined timepoints.

To determine aclarubicin’s mechanism of action in PDAC, we investigated the ability of the different drugs to activate mechanisms of cellular apoptosis. All cell lines were exposed to doxorubicin, gemcitabine or aclarubicin and analyzed for apoptosis induction by poly (ADP-ribose) polymerase. In all cell lines aclarubicin demonstrated to have an apoptotic effect by the induction of cleaved PARP (Fig. 4). In line with Pang *et al*. [20], the induction of apoptosis by aclarubicin, determined by PARP cleavage, induces a cytotoxic effect. The other drug compounds, doxorubicin and gemcitabine, do not induce apoptotic effects in each cell line highlighting the potent effect of aclarubicin in regard to PDAC.

## Discussion

To date, PDAC remains unresponsive to treatment with chemotherapeutic compounds [27]. Although gemcitabine has been the cornerstone of PDAC treatment for many years, this treatment has very limited efficacy. Recently, combinations of different chemotherapeutic drugs have been tested for PDAC, which only slightly improving the overall survival of PDAC patients [28]. Furthermore, the increased survival rates with combination treatments comes at the cost of severe side effects. These observations emphasize the need for novel active agents that target pancreatic cancer biology, and which can be used as monotherapy or in combination regimens with better toxicity profiles. The use of traditional Topo IIα inhibitors as combinatorial chemotherapy for PDAC has shown moderate clinical activity [29–32]. However, aclarubicin has not been extensively tested in PDAC tumors. Aclarubicin is an unusual anthracycline that does not generate DNA breaks but creates chromatin damage by evicting histones at defined sites, effectively acting as an epigenetic modifier [19]. Since aclarubicin is considerably less toxic (especially less cardiotoxic) than classical anthracyclines like doxo- or daunorubicin, it can be used at higher dose or for a longer time period [23]. This defines aclarubicin as an interesting drug to test (preclinically). In the present study, we aimed to evaluate the in vitro cytotoxic effect of the standard-of-care drug monotherapies: 5-FU, irinotecan, and oxaliplatin the different drugs that make up FOLFIRINOX and gemcitabine and compare their effect to doxorubicin, clinically the most used Topo IIα inhibitor; and aclarubicin, a doxorubicin analog and Topo IIα inhibitor, in experimental PDAC cancer cell lines. Aclarubicin had a superior anticancer effect in the majority of the cell lines tested. The IC_50_ of aclarubicin was at least 20–30 times lower than the standard peak serum concentration (6 µM) in patients under normal treatment conditions. This suggests that effective treatment of PDAC patients with aclarubicin is feasible at low dosages. Intriguingly, gemcitabine, the most commonly used monotherapy, had variable responses between cell lines but was never as cytotoxic as aclarubicin. Strikingly, the combination of aclarubicin and gemcitabine augmented its cytotoxic effect, emphasizing the possible synergy between both drugs. This effect might be explained by the different mechanisms of action of these drugs. Gemcitabine inhibits DNA synthesis whilst aclarubicin induces chromatin damage by selectively evicting histones marked with H3K27me3 thereby inducing classical apoptosis by means of PARP cleavage as also demonstrated by our western blot results [19]. H3K27me3 is introduced by the Enhancer of Zeste homolog-2 (EZH2) which is overexpressed in PDAC [33,34]. EZH2 inhibitors like 3-Deazaneplanocin A have shown strong synergy with gemcitabine in assays with PDAC cells [35]. But, while EZH2 inhibitors may only have a moderate effect on the epigenetic profiles of cancer cells, aclarubicin can produce dramatic alterations, making it a promising compound for the treatment of PDAC, particularly in a combinatorial setting.

These preclinical results warrant further clinical testing of aclarubicin in patients with PDAC. Since aclarubicin is already clinically approved and the side effects and treatment schemes known, the next step in testing this compound in PDAC patients could be made swiftly. However, the biodistribution of aclarubicin is currently not known and further studies are required to determine its efficiency in reaching solid tumors. Despite this, our data suggest that aclarubicin may constitute a new treatment option for PDAC patients.

In conclusion, aclarubicin is an effective drug in PDAC cell lines and low-passage PDAC cells with IC_50_ values are around 20–30 times lower than standard peak serum concentrations in human patients. Response to gemcitabine and doxorubicin remains variable between the different cell lines in vitro. Furthermore, the combination of gemcitabine and aclarubicin at low concentrations outperform gemcitabine as monotherapy in our experimental setting. These findings provide a strong rationale for considering aclarubicin with a known favorable adverse event profile as a possible new backbone of combination regimens in patients with pancreatic cancer.

## Acknowledgements

This work was supported by the European Commission H2020 MSCA-ETN grant under proposal number 675743 (project acronym: ISPIC), the Institute for Chemical Immunology (to J.J.N.) and is part of ONCODE Institute, which is partly financed by the Dutch Cancer Society.

All authors contributed to the study conception and design. Material preparation, data collection and analysis were performed by T.P.B., S.Y.v.d.Z., J.D.H.v.E., B.A.B., and M.v.d.P. The manuscript was written by T.P.B. and S.Y.v.d.Z. under supervision of AV and JN with input of all authors.

All data supporting the findings of this study are included in the main text (and supplemental data file).

### Conflicts of interest

J.J.N. is a shareholder in NIHM that aims to produce aclarubicin for clinical use. For the remaining authors, there are no conflicts of interest.
